# Application of super-resolution and correlative double sampling in cryo-electron microscopy†

**DOI:** 10.1039/d2fd00049k

**Published:** 2022-04-22

**Authors:** Yuewen Sheng, Peter J. Harrison, Vinod Vogirala, Zhengyi Yang, Claire Strain-Damerell, Thomas Frosio, Benjamin A. Himes, C. Alistair Siebert, Peijun Zhang, Daniel K. Clare

**Affiliations:** a Diamond Light Source Harwell Science and Innovation Campus Didcot OX11 0DE UK daniel.clare@diamond.ac.uk; b RCaH Harwell Science and Innovation Campus Didcot OX11 0DE UK; c Howard Hughes Medical Institute, RNA Therapeutics Institute, University of Massachusetts Medical School Worcester MA USA; d Division of Structural Biology, Wellcome Trust Centre for Human Genetics, University of Oxford Oxford OX3 7BN UK

## Abstract

Developments in cryo-EM have allowed atomic or near-atomic resolution structure determination to become routine in single particle analysis (SPA). However, near-atomic resolution structures determined using cryo-electron tomography and sub-tomogram averaging (cryo-ET STA) are much less routine. In this paper, we show that collecting cryo-ET STA data using the same conditions as SPA, with both correlated double sampling (CDS) and the super-resolution mode, allowed apoferritin to be reconstructed out to the physical Nyquist frequency of the images. Even with just two tilt series, STA yields an apoferritin map at 2.9 Å resolution. These results highlight the exciting potential of cryo-ET STA in the future of protein structure determination. While processing SPA data recorded in super-resolution mode may yield structures surpassing the physical Nyquist limit, processing cryo-ET STA data in the super-resolution mode gave no additional resolution benefit. We further show that collecting SPA data in the super-resolution mode, with CDS activated, reduces the estimated *B*-factor, leading to a reduction in the number of particles required to reach a target resolution without compromising the data size on disk and the area imaged in SerialEM. However, collecting SPA data in CDS does reduce throughput, given that a similar resolution structure, with a slightly larger *B*-factor, is achievable with optimised parameters for speed in EPU (without CDS).

## Abstract 2

A comparison of STA and SPA processed data acquired with/without super-resolution imaging mode enabled.

## Graphical abstract

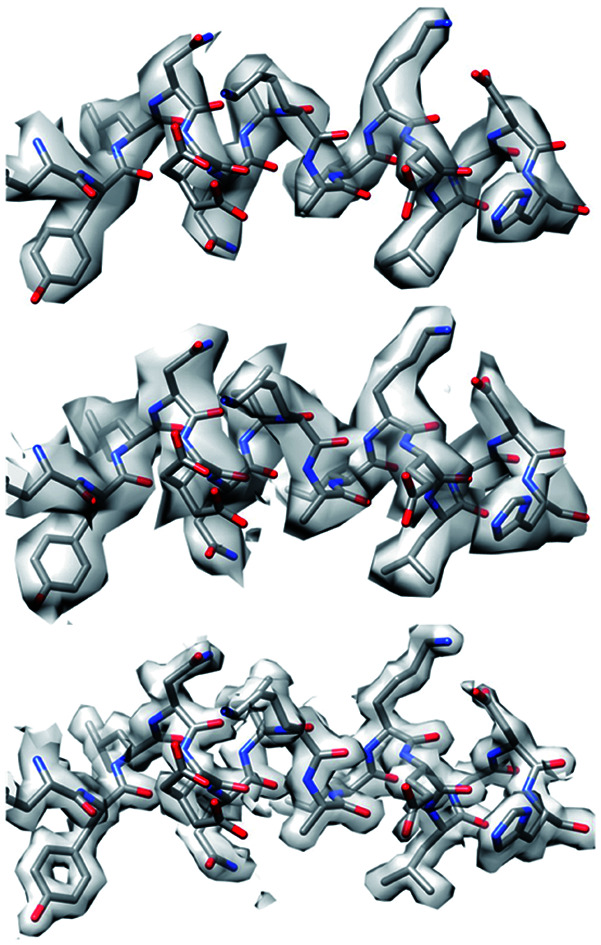

## Introduction

Cryo-electron microscopy (Cryo-EM) has now established itself as one of the main structural biology techniques for high-resolution structure determination, in some cases producing maps with atomic resolution.^1–6^ The growing success of cryo-EM in structural biology is due to several factors. These include technical improvements to the microscopes, automated data collection, data processing software, and primarily the use of direct electron detectors (DEDs). The recording speed of DEDs enables post-acquisition correction for sample motion. Additionally, DEDs have a detective quantum efficiency (DQE) that is much higher than those of either film or CCD cameras,^7^ allowing for increased information transfer at both low and high spatial frequencies. Some DEDs can acquire in a super-resolution (SR) mode, in which the physical pixel area is quartered in software. The SR mode has proven to be effective in the previous generation of detectors (Gatan K2-summit) and has been incorporated in the current generation of detectors (Gatan K3 and Falcon 4). However, most K2 data collections, performed with EPU (ThermoFisher’s single-particle data collection software), do not employ the SR mode as this directly compromises data collection speed. This contrasts with SerialEM,^8^ where no significant speed penalty is observed, and the SR mode is routinely used. This issue of lower data collection rates in the SR mode compared to the non-SR mode with EPU persists today and often requires the user to make a choice at the start of their data collection between acquisition speed or optimal detector performance. The limitation arises with non-SR movies in EPU as EPU does not consider aliasing when going from SR to non-SR and therefore the noise at higher spatial frequencies is increased (https://forum.scilifelab.se/t/some-timing-tests-of-the-gatan-k3/139).

The Gatan K3 detector permits a higher beam current and, hence, a shorter total exposure time than the K2 due to its higher internal frame rate of 1500 fps *vs.* 400 fps, respectively. The increased frame rate reduces coincidence loss, which otherwise reduces the DQE of counting detectors.^9^ The K3 detector chip is also 50% larger than the K2, allowing larger areas to be imaged per tomogram collected, effectively increasing throughput, which is important for tomography applications. In addition, the K3 detector includes correlated double sampling (CDS), in which the voltage of all the pixels is read before and after each acquisition, thus suppressing analogue noise from the detector.^10^ However, due to the two read cycles, the effective internal frame rate of the detector is reduced by half to 750 fps, consequently reducing the beam currents that can be used (7–8 e^−^/p/s *vs.* 15 e^−^/p/s) and, thus, increasing exposure times. The benefit of CDS over non-CDS is a higher DQE, which has been demonstrated by acquiring higher resolution structures of apoferritin when using CDS.^10^ However, it still remains to be demonstrated if the expected beneficial effect of noise reduction when using CDS will have any significant effect on cryo-electron tomography (cryo-ET) and sub-tomogram averaging (STA). In this manuscript, we have tried to assess the effect of using CDS for cryo-ET STA data collection in combination with the SR mode using a test specimen, apoferritin.

Here, we show that a combination of both SR and CDS during data collection has allowed reconstructions of apoferritin to be obtained at a 2.8 Å resolution but this is further improved to better than a 2 Å resolution when processed with SR in SPA, significantly surpassing the Nyquist frequency of the physical pixel size of 1.356 Å (up to 140% of Nyquist), similar to that previously reported by ref. 10. In cryo-ET STA, where data was collected using the same imaging conditions as SPA, on the same grid, we were also able to reconstruct apoferritin to a 2.8 Å resolution. However, processing with SR and CDS did not result in further improvements, suggesting that the benefit of SR and CDS in tomography may not be as apparent as it is for SPA.

## Results and discussion

The initial goal of the study was to compare cryo-ET STA and SPA data collected on the same apoferritin grid, using the same imaging conditions. A further aim was to use apoferritin to accurately calibrate the pixel size of the new K3 detector installed at eBIC^11^ at several commonly used magnifications. To compare cryo-ET STA and SPA, we decided to use a nominal magnification of 64k in EFTEM, equivalent to 1.34 Å per pixel (SerialEM calibration), because at the time of data collection (March 2020), there were no published cryo-ET STA structures at better than 3 Å. In addition, due to issues observed with gain referencing on the K3 in non-SR mode for cryo-ET, all the data were collected using the CDS mode. The CDS mode reduces the detector noise and, therefore, should significantly help with cryo-ET STA data processing. A confounding issue was a requirement to remove the first movie frame of each tilt image due to a Gatan software bug (that has subsequently been fixed in the latest version). An identical counting rate was used in both cryo-ET STA and SPA data collections in order to facilitate the comparison. SerialEM was the only software of choice as both cryo-ET, using a dose-symmetric tilt scheme, and image/beam shift SPA data collections are possible (subsequently, ThermoFisher has implemented the dose symmetric scheme into their tomography software).

### Cryo-ET STA of apoferritin at 64k

**Fig. 1:**
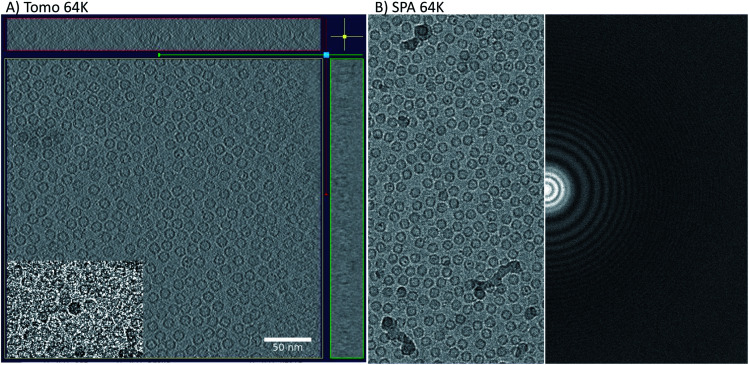
Tomography and SPA raw data. The left side of the figure shows a 3D reconstruction from one of the apoferritin tilt series displayed in *xy* (middle), *xz* (top), and *yz* (A). All three display orientations contain five sections averaged to improve contrast. The inset in the central panel is a raw image from a 0-degree tilt. The right-hand side shows a motion-corrected sum of one of the single-particle movies (left half) and its power spectrum (B).

**Fig. 2:**
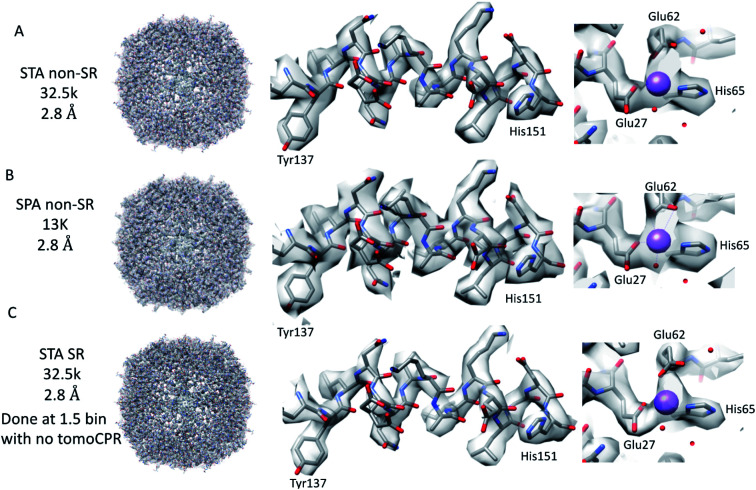
Comparison of tomography and SPA structures. The non-SR 64k STA structure of apoferritin (top row (A)) with the equivalent SPA apoferritin structure (middle row (B)) and the SR apoferritin STA structure (bottom row (C)). Each of the panels is arranged with the whole structure on the left, a helix with residues 137–151 shown in the middle, and the sodium binding site shown on the right. The individual panels were generated using Chimera.^29^

Firstly, 53 dose-symmetric tilt series were collected at a pixel size of 1.34 Å (subsequently, an apoferritin calibrated pixel size of 1.356 Å). These data were reconstructed in IMOD (Fig. 1A), and the 27 closest to focus tilt series were processed further with emClarity^22^ (excluding tomograms at −3.5 micron defocus, leaving only those at −1.5 and −2.5 micron defocuses). From these 27-tilt series, 32.5k sub-tomograms were picked with template matching, aligned, and averaged, giving a resolution of 2.8 Å in the final reconstruction (Fig. 2A and ESI Fig. 1C and 2C†). At this resolution, most side chains were resolved, as well as the sodium binding site (Fig. 2A). The structure determined in this study shows that even with cryo-ET STA data, the K3 operated in the CDS mode can preserve high-frequency information near to or at the Nyquist frequency of the images (∼96% of the Nyquist frequency at 2.68 Å). Interestingly, with just two tilt series and 2.4k sub-tomograms, it was possible to achieve close to a 2.9 Å resolution (ESI Fig. 1A and 2A†). With six-tilt series, equivalent to 7.2k sub-tomograms, the reconstruction resolution did not improve significantly. However, the overall quality of the density did when compared to the reconstruction from the two-tilt series (ESI Fig. 1B and 2B†). This six-tilt series data set was also processed independently, and it was possible to achieve a slight improvement in resolution to 2.8 Å from 2.9 Å. This dataset was subsequently deposited in the EMPIAR database (EMPIAR-10787) as a training dataset for the emClarity protocol paper.^12^ The overall quality of the density improved further with the 27-tilt series. However, as the resolution was close to Nyquist, it did not improve any further and stayed at just below 2.8 Å, as measured by Fourier shell correlation (FSC).

Because we saw no significant improvement in the resolution as we increased the number of sub-tomograms included in the final map, we decided to test whether interpolation artefacts were limiting us. To test this hypothesis, we increased the effective pixel sampling rate by processing the 27-tilt series data in SR using two different procedures, owing to the limited GPU memory available for handling SR tomography data. In the first, the post-motion-corrected tilt series were aligned at full SR in IMOD (with a pixel size of 0.67 Å), and then Fourier cropped by 1.5 for the final rounds of alignment and reconstruction in emClarity (with a pixel size of 1 Å). In the second, after the motion correction, the tilt series were Fourier cropped by 1.5 and processed with IMOD and emClarity alignment and reconstruction (with a pixel size of 1 Å). We had to use both methods as the tilt-series refinement algorithm in emClarity, tomoCPR, reconstructs a full tomogram, which requires more memory at SR than available on our current hardware.

Using both procedures, we measured resolutions beyond the physical Nyquist limits of 2.43 Å and 2.35 Å (Fig. 2C and ESI Fig. 1D and 2D†). However, upon closer inspection of the density, it was clear that the features of the SR maps did not reflect the increased resolution reported by the FSC (ESI Fig. 2†). This was evident when the SR maps were compared to the non-SR map from emClarity, a SPA reconstruction at a resolution of 2.5 Å (collected at the same magnification, in SR, as the tomography data), or the 2.3 Å cryo-ET STA structure at a smaller pixel size from a previous study^13^ (ESI Fig. 4†). This was further confirmed when the reconstruction was calculated directly from the original projections rather than as a sub-tomogram average using the “reconstruct” option in emClarity. Here, the resolution measured for the maps was closer to 2.8 Å. This apparent inflation in the resolution suggests that our attempt to refine the SR data using these external procedures circumvented the safeguards implemented in emClarity to prevent over-fitting. For completeness, we also applied the reconstruct option to both the two-tilt series and six-tilt series STA data sets and got resolutions of 3.6 Å and 3 Å, respectively. This compares to 2.9 Å estimated for both data sets with the standard STA averaging and FSC estimation in emClarity. The resolution estimates for the two-, six- and 27-tilt series data sets, using the reconstruct option, now scale with the number of sub-tomograms (2.4k giving 3.6 Å, 7.2k giving 3 Å and 32.5k giving 2.8 Å) with a calculated *B*-factor of 28 Å^2^ which compares favourably to those calculated for the SPA data sets later in the Results section.

Despite the limitations on image processing imposed by the size of the SR data, it is still impressive that information to near the physical Nyquist limit can be preserved. At this magnification, the K3 images had a field of view of 0.43 μm^2^ when referring to the specimen, with each tomogram containing over 1200 apoferritin particles. As most real-world STA projects do not reach or exceed 3 Å resolution, 64k magnification may be the sweet spot for data collection, maximising the area imaged while maintaining the potential STA resolution. It would also be interesting to test if the SR mode, at lower magnifications, would make a difference with apoferritin.

### SPA of apoferritin at 64k and comparison to the STA structure

**Fig. 3:**
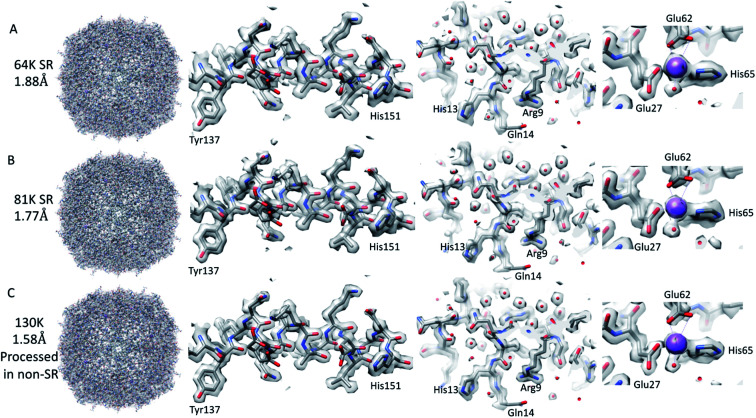
SPA reconstructions. The SR 64k SPA structure of apoferritin (top row (A)), the SR 81k SPA apoferritin structure (middle row (B)), and the 130k SPA apoferritin structure (bottom row (C); collected in SR but processed in non-SR). Each of the panels is arranged with the whole structure on the left, a helix with residues 137–151 shown in the middle left, a slice through the structure showing the water molecules in the middle right, and the sodium binding site shown on the right. The individual panels were generated using Chimera.

The resolution of the initial SPA dataset collected at a nominal magnification of 64k was at to the Nyquist frequency of the images before ctf refinement and particle polishing in Relion, even with 27k particles (ESI Fig. 5A† – 55k particles in the structure shown). Therefore, the data was processed in the SR mode to check if the information extended beyond the Nyquist limits. From the power spectra of the motion-corrected movies, it was possible to see the graphene reflection at 2.1 Å (ESI Fig. 6† top row). Prior to CTF refinement and polishing, a 2.3 Å resolution map could be attained (220k particles); post CTF refinement, this extended to 2 Å and post polishing, this was 1.92 Å (220k). At this point, all particles were combined, giving a total of 320k, and higher-order aberrations were refined, giving a final reconstruction at 1.87 Å (Fig. 3A and ESI Fig. 3A and Table 1†). This is approximately 140% of the physical Nyquist limit (based on an apoferritin calibrated pixel size of 1.356 Å). This is very similar to the values reported by two other recent publications.^10,14^

In contrast to the SPA structure, the resolution of the apoferritin STA structure, even when processed in SR, seemed to be limited to around 2.8 Å (32.5k sub-tomograms from the 27-tilt series, Fig. 2C). For the SPA data set collected at a magnification of 64k, it was possible to achieve 2.8 Å, prior to the ctf refinement step, with around 13k particles. This structure was almost identical to that of the STA structure (Fig. 2B and C and ESI Fig. 4A–D†), and the density comparison between the STA and SPA structures was further improved if Relion was used to sharpen the cisTEM calculated half maps of the STA structure (ESI Fig. 4C and D†). Additionally, if we reconstructed the SPA structure from 32.5k particles, the same number of particles as sub-volumes in the 27-tilt STA structure, 2.55 Å could be achieved (ESI Fig. 4E,† again prior to ctf refinement). The 2.8 Å resolution limit we see in our STA is not a hard limit for STA apoferritin structures, as a 2.3 Å resolution has been achieved in a previous publication (ESI Fig. 4F;† ref. 13). However, this structure was done at a higher magnification and with fewer particles, but with 135 tilt series, than the structure determined in this study (105k× ∼0.829 Å/pix *vs.* 64k× 1.356 Å/pix). It is plausible that a better DQE at higher spatial frequencies when increasing the magnification may be required to get to higher resolutions.

### SPA of apoferritin at higher magnifications

We went on to collect data sets at 81k EFTEM and 130k EFTEM (approximately the same pixel size as 64k in SR) on the same apoferritin grid. At 81k, we saw the same behaviour as with 64k, in that we could get to the Nyquist frequency with no CTF refinement or Bayesian polishing (ESI Fig. 5B and Table 1†). Once the additional refinements were done, we reached 1.77 Å (Fig. 3B and ESI Fig. 3B†), which was 120% of the physical Nyquist frequency (based on a calibrated pixel size of 1.078 Å and 250k particles). This is still impressive, but shows that it gets more difficult to go past the physical Nyquist frequency as you go to higher resolutions and suggests that something other than the detector DQE may be the main barrier. If we treated the data collected at 130k, where the physical pixel size was similar to that of the 64k data in SR (0.677 *versus* 0.651), then we achieved a slightly higher resolution, but with more particles, of 1.74 Å (Table 1), which was slightly concerning as we expected to get a higher resolution at this magnification. A potential explanation for this was that this data set was initially collected for accurate pixel calibration and initial detector testing and, as such, the defocus range was not optimised for high-resolution structure determination (the defocus range used was −0.5 to −2 microns).

**Table 1:** Resolution estimation at the different refinement steps in Relion

Magnification	Number of particles	Initial resolution (Å)	Resolution post CTF refinement (Å)	Resolution post Bayesian polishing (Å)	Resolution post BT, 3 & 4 order + mag (Å)
64k	320k	2.68 (2.3 SR)	2	1.9	1.87
81k	240k	2.12 (2.02 SR)	1.93	1.89	1.77
130k	470k	1.92	1.81	1.79	1.74^a^
130k	250k	1.78	1.69	1.67	1.58
Max defocus 1.6 μm & large box					
130k-EPU	500k	2.07	1.81	1.75	1.59

a For the 130k magnification that contained 470k particles, any defocus over 1.5 μM was removed as angstrom test, leaving 170k particles in the standard box size, and it still reached 1.74 Å.

We, therefore, reprocessed the 130k dataset, limiting the maximum defocus to 1.5 μm (0.5–1.5 μm), reducing the number of particles to 240k from 470k and also increasing the box size such that we removed any potential CTF aliasing (estimated using the online resource by Takanori Nakane, https://3dem.github.io/relion/ctf.html). This increased the attainable resolution from 1.74 to 1.58 Å (Fig. 3C, Table 1 and ESI Fig. 3C†). This was still only 83% of the physical Nyquist frequency, which was lower than anticipated, based on the results at the lower magnifications, which both exceeded the Nyquist frequency. One potential cause of this resolution limit may have been that these data were collected with the beam tilt corrected image/beam shift method, and the beam tilt correction may not have been accurate enough. To test this, images with the equivalent image/beam shift collected from each stage position (49 positions – 9 × 5 matrix) were treated as individual optics groups in Relion, and the beam-tilt was estimated and corrected. The data was then reconstructed, and the resolution of the final map was identical (tested only for the 130k data set). It may be that this is the maximum achievable resolution at this magnification, as all the structures of apoferritin resolved at better than 1. 6 Å have been done with higher magnifications (smaller pixel sizes), suggesting that the increased DQE of the detectors at higher spatial frequencies when the magnification is increased are required to get past this resolution^3–6,10,13^ (ESI Table 1†). In order to achieve a high-resolution with apoferritin, both the sample preparation and grid preparation are very important factors but, interestingly, the specific species of the apoferritin sequence used does not seems to be a major factor as both human and mouse homologues have generated sub 1.6 Å structures (ESI Table 1†).

The resolution achievable at lower magnifications using SR suggests that it may be more efficient to collect data at these lower magnifications. However, when factoring in the higher beam dose rate that can be used at higher magnifications, both the time it took and the total area of the hole imaged were similar to those at lower magnifications, similar to that reported in other studies.^15^ For example, at 64k with two movies per hole, the total area imaged was equivalent to 0.868 μm^2^ with a collection time including image delays of 17 seconds. At 81k, this equates to 0.822 μm^2^ and 16.5 seconds for three movies, and at 130k to 0.835 μm^2^ and 15 seconds for five movies. With the increased DQE at higher relative spatial frequencies when collecting at higher magnifications, as reflected by the *B*-factor calculated for 64k of 77.5 Å^2^ compared to that at 130k of 55.9 Å^2^, the only potential benefit of collecting at lower magnifications is that fewer images are required. This may have been significant when data were collected at full bit depth due to the file sizes on disk, but with most data collections using low bit depth formats and compression, this is not as important as it once was.

In a further comparison, conducted after the initial data collection, we also assessed the effect of CDS, as well as the data collection speed, on the maximum resolution achievable at 130k with the K3 detector. For this additional dataset, we took advantage of recent fringe free illumination (FFI) and aberration-free image/beam shift (AFIS) upgrades to the microscopes at eBIC in order to maximise throughput within EPU.^15^ Another benefit of FFI, in particular, was that we were now able to collect 12 movies per hole, meaning that we effectively imaged an area of 2 μm^2^ per 2 μm hole (max 3.14 μm^2^) compared to 0.835 μm^2^ with no FFI. As this dataset was collected in EPU, we also had to acquire the data at SR bin 2 (https://forum.scilifelab.se/t/some-timing-tests-of-the-gatan-k3/139) for maximum throughput. This should reduce the DQE at high spatial frequencies as the averaging during movie collection in EPU does not compensate for aliasing. However, this, in combination with the recent microscope upgrades and the use of R2/2 grids on graphene, allowed 12 movies per hole to be collected, giving a data collection rate of over 1000 movies per hour. From these data, processed in the same way as the original data collected in CDS, we were able to achieve a 1.59 Å resolution (ESI Fig. 3D†). However, this was from 500k particles, which was double that in the final 130k CDS structure, and with the equivalent number of particles we could only reach 1.66 Å. The collection of the 130k-EPU data set was significantly faster than with CDS (∼3 times faster) while maintaining an almost equivalent resolution and map features (ESI Fig. 7†). Another interesting observation is that the *B*-factor calculated from this structure was only marginally worse than that calculated for the CDS which was SR binned with anti-aliasing in MotionCor2, at 57.1 compared to 55.9. This data set did not require the same large box size as with the 130k as the defocus range was better optimised for high-resolution data collection (0.3–1.6 μm, Table 2).

**Table 2:** Data collection parameters for the SPA data set reconstructed in this study

Magnification	C2 (μm)	Counting rate/dose rate (e^−^/pix and e^−^/Å^2^/s)	Exposure time (s)	Total dose e^−^/Å^2^	Frames per movie	Number of movies	Defocus range (μm)
64k	50	7.6–4.2	8	33.6	40	450	0.9–2.5
81k	50	7.2–6.4	5	32	40	1107	0.8–2.4
130k	50	5.8–13.7	2.5	34	40	2295	0.5–2
130k-EPU	50	12.4–29.3	1	29.3	40	9049	0.4–1.6

### Analysis of the resolved water in apoferritin

In the 6z6u structure used as the starting model for structure refinement, there were 139 water molecules present. The density for most of these water molecules was present at one sigma (see Methods), with a maximum of 10 missing in the 64 and 81k sub 2 Å maps. The quality of the density for the water molecules across the multiple reconstructions was good, with the individual water molecules resolved in all structures at a 2.12 Å resolution and better, with the strongest water molecule density visible as a continuous density at 2.7 Å (Fig. 3 and ESI Fig. 5†). This confirms previous observations that there is high consistency in water molecule positions between different cryo-EM maps (76% based on ref. 4). There were some small variations in the positions of some water molecules when different maps were compared. In addition, not all of the water molecules were in each of the four maps at a 2 Å or better resolution. However, these tended to coincide with the water molecules with the least amount of density at one sigma. If the maps were displayed at two sigma, the number of water molecules missing went from at worst 72 (64k SR map) to at best 53 (130k-EPU map). Three additional waters near the sodium ion were added to the coordinates during the refinement procedure, but this is still well short of the over 300 water molecules contained in the crystal structure of human apoferritin (2chi).^16^

## Conclusion

The K3 detector operated in the CDS mode can record high-quality tilt series that allow STA reconstructions near the physical Nyquist limit of the images at a magnification of 64k. However, we were not able to extend the resolution any further with STA when the tomograms were processed with SR. This was in stark contrast to the SPA data that we collected using the same sample, grid, and imaging conditions, which were able to exceed the physical Nyquist limit by 140%. Even if the reduced number of sub-tomograms used in the STA analysis is factored in, 2.5 to 2.6 Å would still be expected to be achieved with the equivalent number of particles in SPA (32.5k particles, the same as in STA, in SPA before ctf refinement in Relion). With the additional information in the sub-tomograms, better than this may be expected in an ideal situation, as each sub-tomogram has effectively 41 individual projections, but at a lower dose than a SPA projection, and with variable levels of radiation damage. However, with the complexity of STA data processing, it may not be a surprise that the STA map is poorer than the equivalent SPA map. Another consideration is that we may need to use higher magnification to achieve an increased resolution, as shown in a previous study,^13^ with the only downside being the reduction in sample area imaged. On the other hand, the results indicate that there is still room for further improvement in cryo-ET STA.

For the SPA data collection, it is clear that collecting at SR can be beneficial at lower magnifications and that this effect is reduced as the magnification and or attempted reconstruction resolution is increased. With the number of movies per hole increasing and the exposure times per movie decreasing, the only advantage of using lower magnifications is that the total number of movies collected would be smaller and therefore take up less space on disk. The only additional benefit may be that for samples with sparse particle distribution, the lower magnification may allow better CTF estimation. The use of CDS does improve the quality of the reconstructions achievable, but at the expense of the increased exposure times required. It may be that this can be offset by collecting more particles using settings for higher throughputs (there should be a cross-over point based on the *B*-factor). CDS may be more important for smaller particles where any reduction in noise could be crucial to solving the structure. With the constant progress in data collection strategies, it is clear that even data collections optimised for speed (FFI, AFIS, and super-resolution bin 2) can generate high-resolution structures with *B*-factors similar to that of data collections optimised more for movie quality.

## Methods

### Apoferritin preparation

The human apoferritin plasmid (LF2422) was provided by the Protex facility at the University of Leicester, specifically Louise Fairall and Christos Savva. This was transformed into Rosetta (DE3)-pRARE-2. Multiple transformant colonies were used to inoculate a 10 mL LB starter culture, supplemented with 100 μg mL^−1^ carbenicillin and 34 μg mL^−1^ chloramphenicol, which was grown for 18 hours at 37 °C. This was then used to inoculate a 1 L AIM TB (Formedium) culture, supplemented with 10 mL glycerol and 100 μg mL^−1^ carbenicillin, grown for 5 hours at 37 °C 200 rpm before reducing to 18 °C for a further 42 hours. The cells were harvested by centrifugation and stored at −80 °C prior to processing. Approximately 30 g of cells were resuspended in 90 mL of phosphate-buffered saline (PBS), supplemented with 0.5% Triton X-100, one complete EDTA-free protease inhibitor tablet, and 1 mM TCEP. Cells were lysed by sonication on ice for an accumulated time of 3 minutes (10 seconds on/10 seconds off) and an amplitude of 35%. The lysate was centrifuged at 50 000×*g* for 30 minutes at 4 °C. The cleared lysate was mixed with 6 mL 50% GST-agarose (Sigma) on a rotary wheel for 1.5 hours at 4 °C. The slurry was applied to a gravity column and washed with 140 mL PBS, supplemented with 0.5% Triton X-100 and 1 mM TCEP. The resin was subsequently washed with 120 mL of gel filtration column (GFC) buffer (50 mM Tris–HCl, pH 7.5, 100 mM NaCl, 1 mM TCEP) and the resin was recovered from the column in an additional 20 mL of GFC buffer. TEV protease was added at a ratio of 10 : 1 (ApoF : TEV) and incubated at 25 °C for 18 hours on a rotary wheel. The slurry was re-applied to the gravity column, and the flow-through, containing ApoF, was collected. The sample was concentrated to ∼3 mL using a 30 kDa MWCO Amicon centrifugal concentrator at 15 °C and passed through a 0.22 μm Millex filter onto a Hiload 16/60 S200 (Cytiva) column, pre-equilibrated in GFC buffer. The peak samples were pooled and concentrated, as above, to ∼5 mg mL^−1^.

### Grid preparation

The apoferritin sample was applied to an in-house graphene-coated Quantifoil R2/2 EM grid (Quantifoil, GmbH Germany) at a concentration of 10 μg mL^−1^ for the grids used in the first SPA and STA data collections. For the later SPA 130k-EPU data set, the apoferritin used was a new batch and was applied at a concentration of 0.5 mg mL^−1^ to the same in-house graphene coated Quantifoils as before. The grids were plunge frozen using a Mark IV Vitrobot (Thermofisher) at 100% humidity, with a temperature of 4 °C and a blot time of 2.5 seconds, leading to a mono-disperse thin layer of apoferritin. The graphene films were grown on a copper sheet (25 μm thickness, Alfa Aesar) in ambient pressure at 1070 °C by chemical vapour deposition and could then be transferred onto various EM grids based on our previous report.^17^

### Tomography data collection

53 tilt series were collected using the same grid, microscope, energy filter, and imaging conditions as for the 64k SPA data set. The tilt series were collected using the tilt series controller in SerialEM^8^ with a dose-symmetric tilt scheme^18^ in 3° increments from 0 to −60/+60 in groups of two tilts per tilt reversal. Each tilt image was collected in the SR mode in compressed tif format as a 10-frame movie with an exposure time of 0.6 seconds, which was equivalent to 2.5 e/Å^2^/tilt (0.25 e per frame), giving a total dose per tilt series of 102 e/Å^2^. The defocus ranges for the tilt series were set to −1, −2 and −3 μm.

### Tomography processing

Using custom scripts from the Zhang lab, the movies for each tilt image were motion-corrected using MotionCor2,^19^ and the first frame from each tilt image movie was removed due to the CDS noise issue, followed by CTF estimation with CTFfind^20^ and re-stacking of the tilt series. CTF determination showed that the tilt series defocuses were closer to 1.4 μm, 2.5 μm, and 3.5 μm, respectively. Each tilt series was then aligned and reconstructed using IMOD,^21^ with patch tracking used as the fine alignment step, as there were no gold fiducial markers in the tilt series. Once reconstructed, the tomograms were split into different batches, with two-tilt series, six-tilt series (data set deposited as part of ref. 12), and 27-tilt series batches (with defocus group 3.5 excluded from this set) used for further processing with emClarity.^22^ The reconstruction from the single particle data set was then used for template matching. The picked sub-volumes were then manually pruned to remove false positives using IMOD, leaving 2.4k, 7.2k and 32.5k for each of the 2, 6 and 27-tilt series, respectively. The parameter files and the processing workflow, similar to that used in this paper, were as previously published.^12^ The number of alignment rounds for each data set were as follows: 14, 18 and 20 for the 2, 6 and 27-tilt series. TomoCPR was performed at bin 4 and bin 2 for all of the 3 data sets processed at the physical pixel size. For the SR STA analysis, only the 27-tilt series data set was used, and during motion correction, the binning was set to either no binning or a bin of approximately 1.5 times, giving 0.651 and 1.05 Å/pixel, respectively. The motion-corrected re-stacked tilt series were then aligned and reconstructed with IMOD and processed with emClarity as before. The two sets used as the results from tomoCPR could only be incorporated into the refined tilt series alignment if the tilt series were binned at least 1.5 times prior to processing in emClarity. This was also the case for the final round of alignment and averaging, which again would only work with a binning of 1.5 (pixel size of 1.05). The final reconstructions were calculated using the cisTEM^23^ option in emClarity with the dose limited to 40 e. The two half maps from cisTEM were also used in Relion for map sharpening and FSC assessment.

### Single particle data collection

The majority of experimental data were collected at SR (11 520 × 8184) in CDS mode using the compressed tif format with image/beam shift data collection in serialEM^8^ on an X-feg equipped G2 Krios (Thermofisher, USA) using the Bio-quantum K3 filter/detector (Gatan/Ametek, UK) with a 20 eV wide energy selecting slit. The only exception to this was the 130k-EPU data set, that was collected using another graphene coated apoferritin grid, on a G3 Krios, recently upgraded with both AFIS and FFI. Additionally, EPU was used to collect the data, with the CDS mode turned off and with SR bin 2 selected in the data acquisition mode, in order to maximise the data collection speed. The different magnification data sets and their corresponding parameters are shown in Table 2 below.

### Single-particle processing

All the data sets were initially processed using a relion_it.py script (https://github.com/3dem/relion/blob/master/scripts/relion_it.py) modified to work on the Diamond Light Source compute cluster. This script runs MotionCor 2,^19^ CTFfind,^20^ crYolo^24^ and 2D classification in Relion 3.1.^25^ Due to the issue with the CDS mode, the first frame of each movie had to be removed. After initial picking, there were 690k (64k mag), 925k (81k mag), and 640k (130k mag), respectively, for the 3 data sets. From these particles, good 2D classes were selected, and the particles extracted for further processing gave 320k, 490k, and 520k particles, respectively, for each of the data sets. From these data sets, 3D classification was performed with the best 3D classes being used for further processing, leaving 320k, 250k, and 470k, respectively. After this, each data set was further processed in the same way with the steps as follows; (1) CTF refinement – defocus and astigmatism, (2) 3D refine masking and post-processing, (3) particle polishing, (4) 3D refine, masking, and post-processing, (5) CTF refinement – defocus, and astigmatism, (6) 3D refine, masking and post-processing, (7) CTF refinement – beam tilt, 3^rd^ and 4^th^ order, (8) CTF refinement – magnification distortion, (9) 3D refine, masking and post processing. Additional processing was performed on the 130k data set, in that a subset of particles, based on defocus values of less than 1.5 μm (leaving 250k particles), were selected post CTF refinement (step 1 above) and the box size that they were extracted in increased from 312 to 600 pixels based on CTF aliasing. All the subsequent steps were the same as for the other data sets. Some additional refinements were tried but no increase in resolution was obtained.

The 130k-EPU data set was processed initially with the relion_it.py script using the same parameters as the above data sets. The two exceptions were that the movie stacks were saved at the physical pixel size of the K3 (5760 × 4092) and that the first frame was retained as the movies were not collected in CDS mode. After initial picking, there were 1 M particles from these particles. Good 2D classes were selected, giving 500k particles for further processing. The 3D classification was not performed with the rest of the processing following the same procedure as described above for the CDS data sets. The 130k-EPU data set was better optimised for defocus than the previous 130k data set, and it was processed in the standard box size (larger box size had no effect).

### Structure refinement

The coordinates from the PDB 6z6u (ref. 3) were docked into the sharpened EM density for the 130k apoferritin structure. The coordinates for a single monomer were then used to cut out the density, at a radius of 4 Å, in order to generate a map with density for only a single monomer. This was then used as the starting point for the refinement of the coordinates with REFMAC *via* the CCP-EM interface.^26,27^ After refinement of the monomer, a number of multiple conformers from the 6z6u structure were removed, and some additional water molecules were added. To refine the subunit interfaces in the apoferritin 24mer, the monomer was docked into the map, and the symmetry-related apoferritin monomers were generated and docked. The apoferritin 24mer was then refined with REFMAC with the local symmetry operator enforced. This structure was then analysed for geometry with molprobity^28^*via* the CCP-EM interface with the statistics shown in Fig. 9 of the ESI.†

Comparison of the water molecule positions between the refined coordinates and the cryo-EM densities was done in Chimera. The sigma value of the map was estimated based on the local zoning of the map around the refined coordinates for a single monomer in Chimera.

### Depositions

The cryoEM density maps for 64k SR, 81k SR, 130k, 130k-EPU, two-tilt series, six-tilt series, 27-tilt series have been deposited in the EMDB with accession numbers EMD-14333, EMD-14335, EMD-14332, EMD-14337, EMD-14350, EMD-14349 and EMD-14348. The coordinates refined from the 130k cryo-EM density were deposited in the PDB with accession code 7R5O.

## Author contributions

D. K. C., C. A. S. and P. Z. conceived of the study and designed the experiments. C. S.-D. expressed and purified the protein, Y. S. and V. V. prepared and screened the grids and D. K. C. and P. J. H. performed the data collection. The majority of the SPA and STA image processing was performed by D. K. C. with contributions from P. J. H., T. F., B. A. H. and Z. Y., Figures were prepared by D. K. C and all authors helped with data analysis and manuscript writing.

## Conflicts of interest

There are no conflicts of interest to declare.

## Supplementary Material

FD-240-D2FD00049K-s001

FD-240-D2FD00049K-s002
