# Supplementary material for: Application of super-resolution and correlative double sampling in cryo-electron microscopy
Source: Faraday Discuss. 2022 Apr 22;240:261–76. doi: 10.1039/d2fd00049k (PMC9642007; doi:10.1039/d2fd00049k)
Supplement: FD-240-D2FD00049K-s001 [file FD-240-D2FD00049K-s001.pdf]

Electronic Supplementary Material (ESI) for Faraday Discussions.
This journal is © The Royal Society of Chemistry 2022

Sup Figure 1

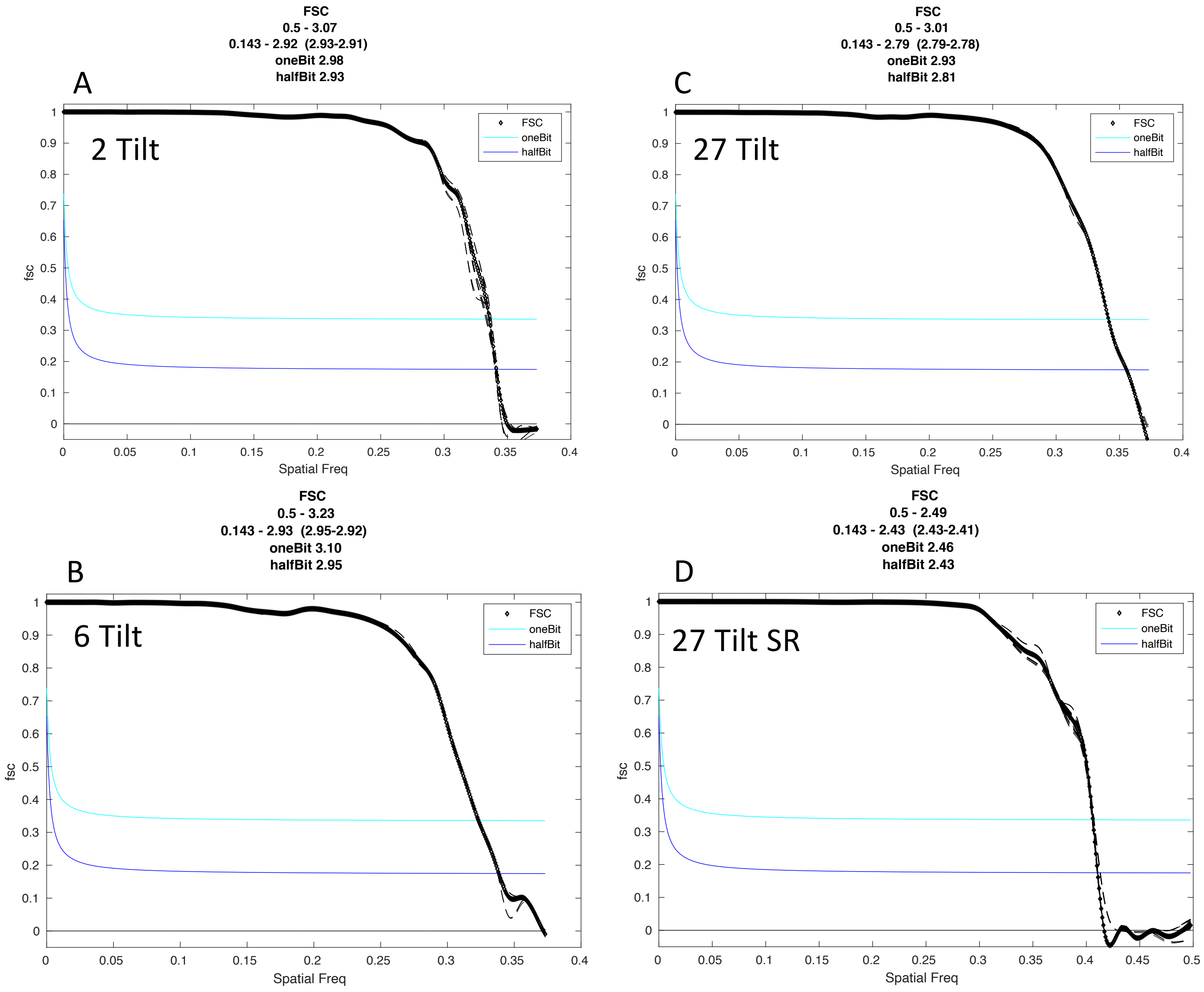

Sup Figure 2

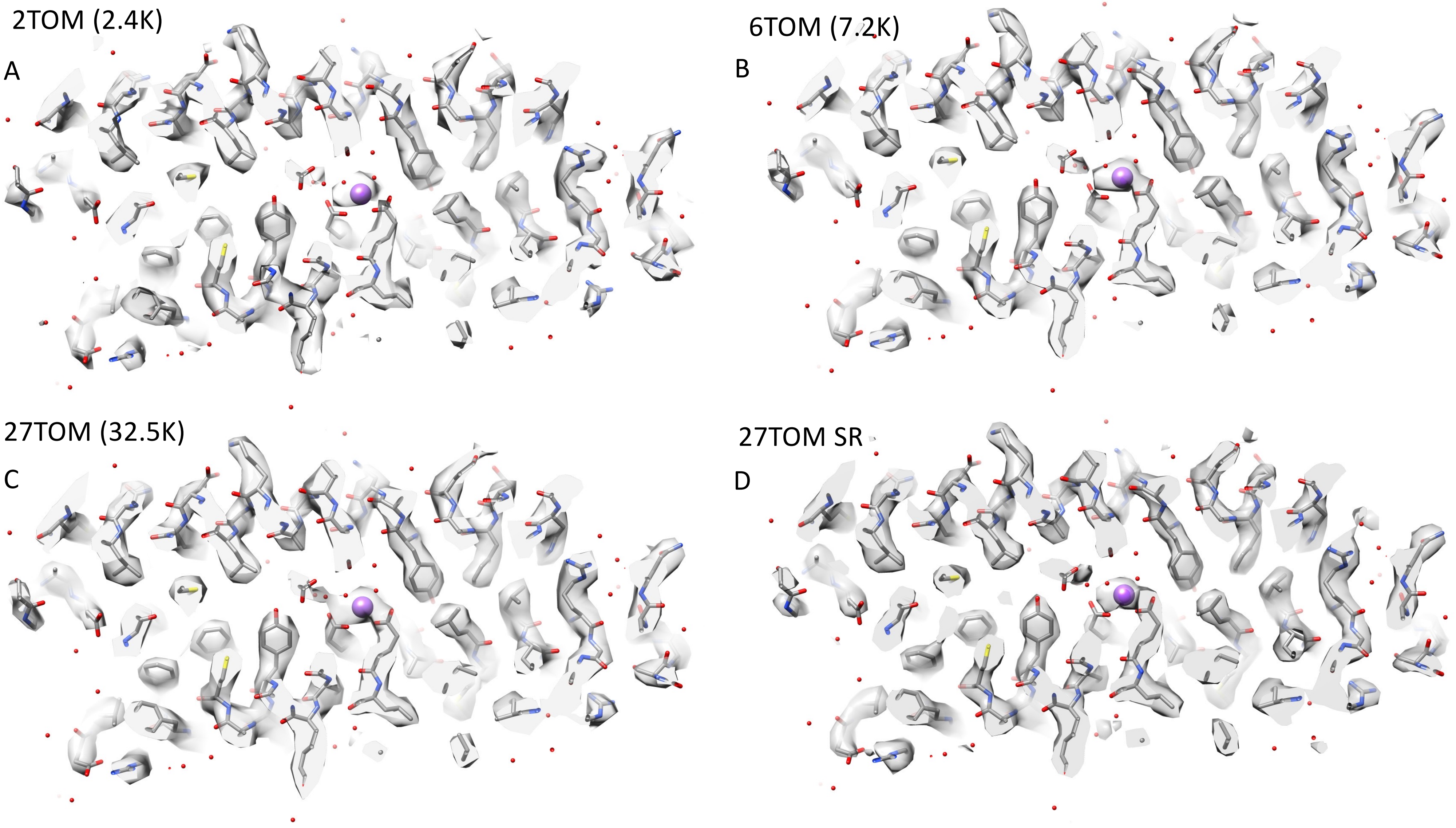

Sup Figure 3   FSC curves for SPA

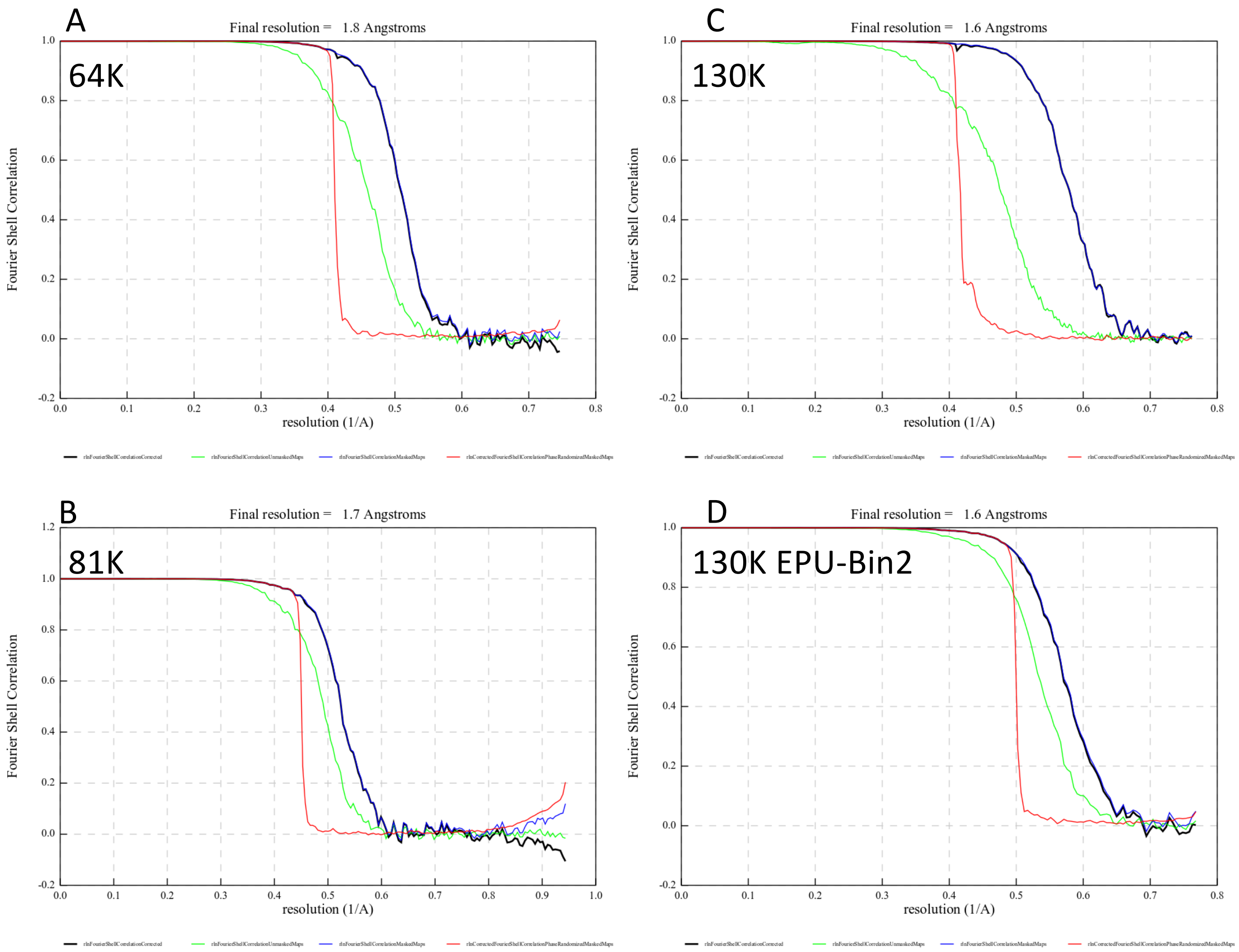

Sup Fig 4 (equivalent resolution)

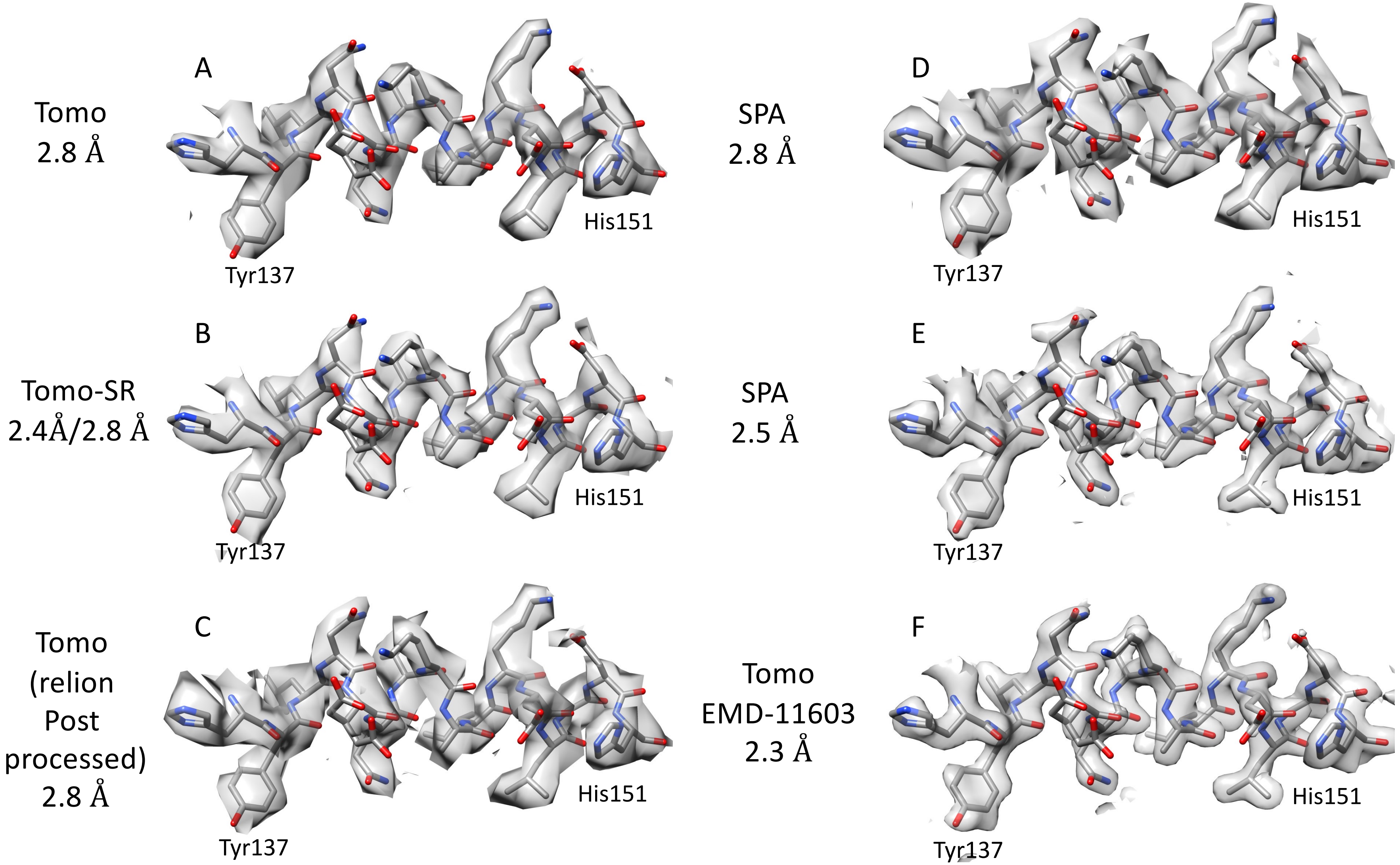

Sup Figure 5

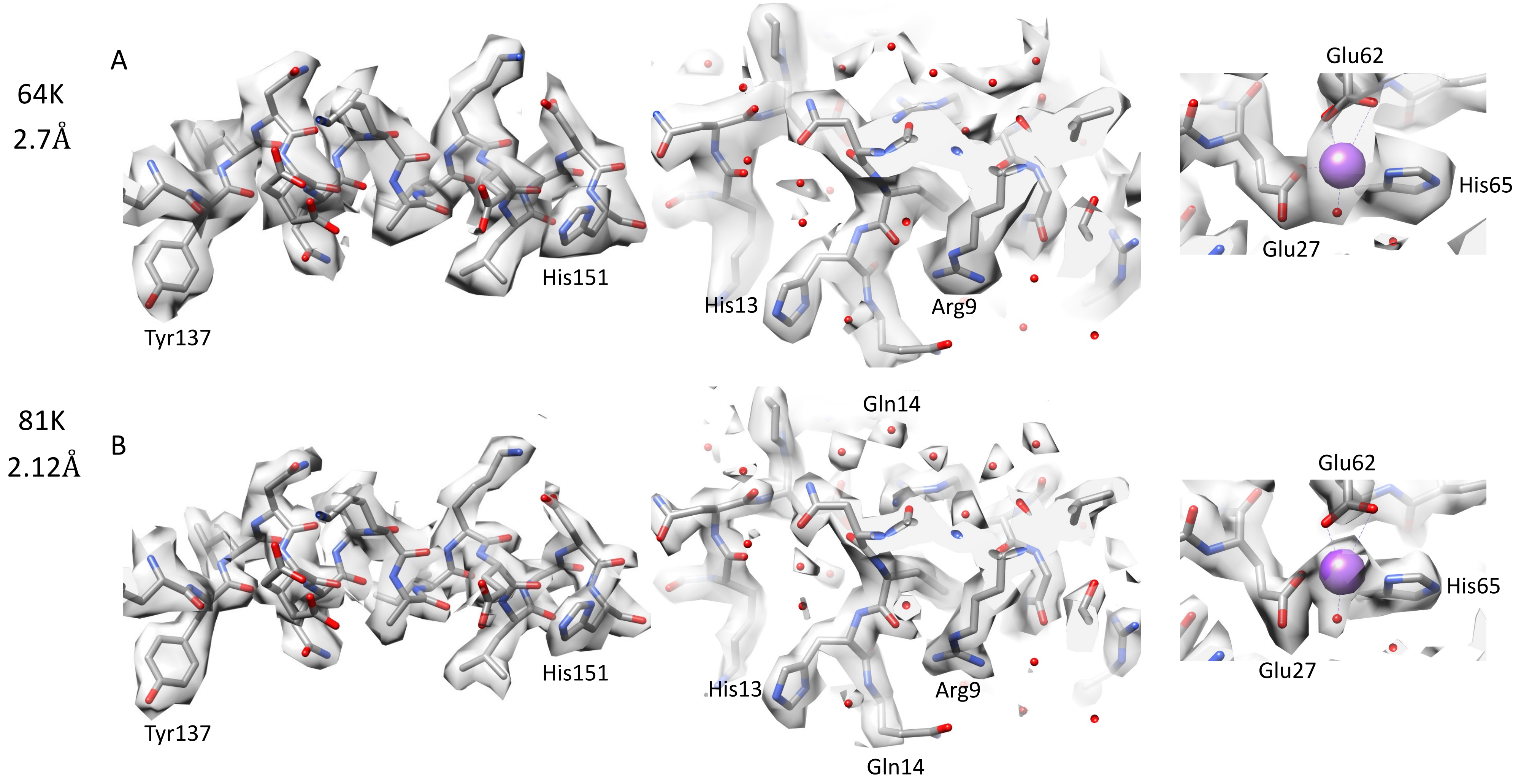

64K
2.7Å
A
Tyr137
His151
His13
Arg9
Glu62
His65
Glu27
81K
2.12Å
B
Gln14
Tyr137
His151
His13
Arg9
Gln14
Glu62
His65
Glu27

Sup Figure 6

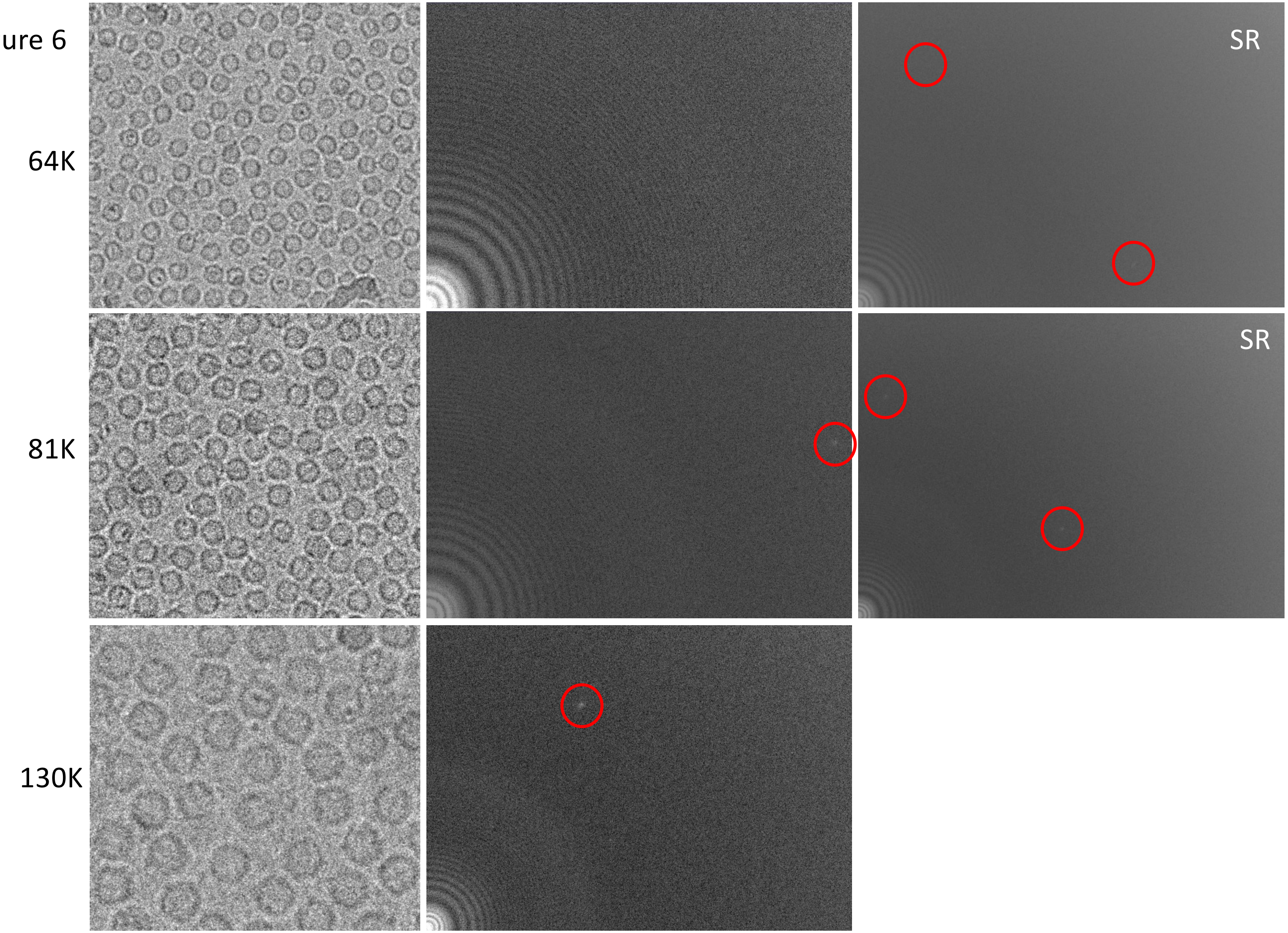

Sup Figure 7

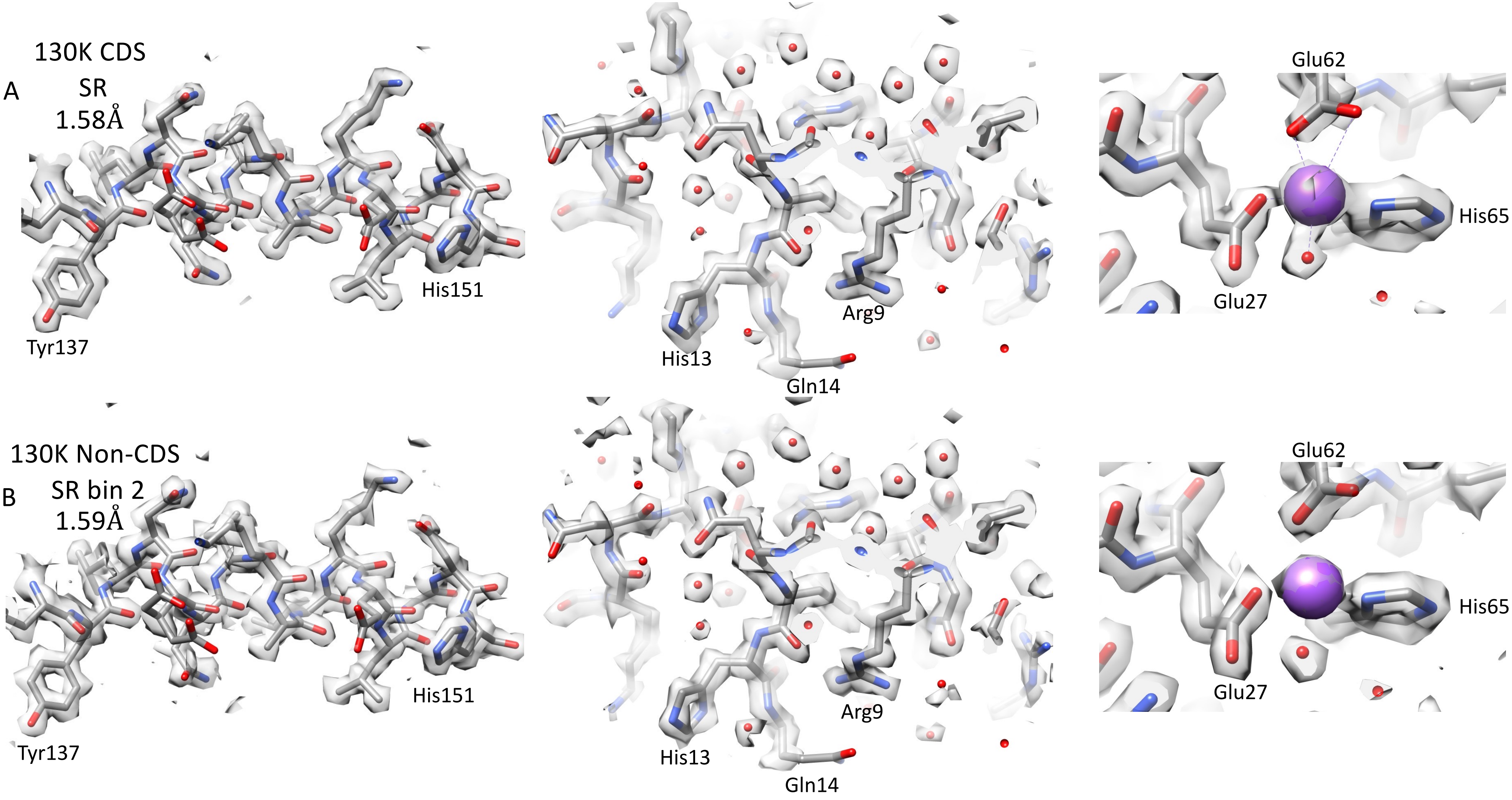
130K CDS
A
SR
1.58Å
Tyr137
His151
His13
Gln14
Arg9
Glu62
His65
Glu27
130K Non-CDS
B
SR bin 2
1.59Å
Tyr137
His151
His13
Gln14
Arg9
Glu62
His65
Glu27

Sup Figure 8

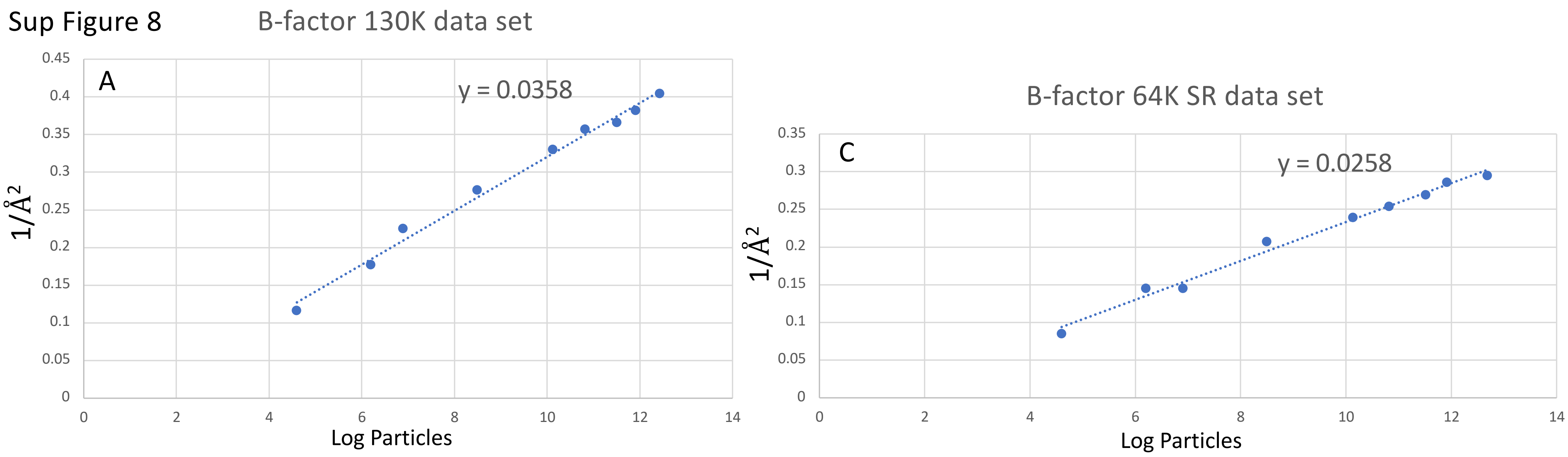

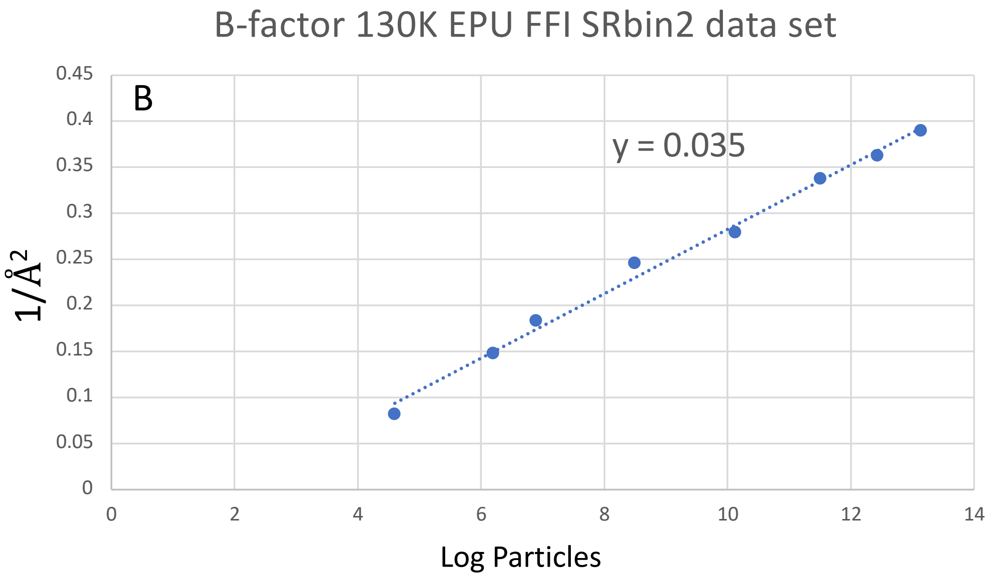

B-factor calculation

2/slope= B-factor in Å^2

64K SR = 77.5 Å^2

130K = 55.9 Å^2

130k-EPU SR bin2= 57.1 Å^2

Sup Figure 9

	Outliers (refined_0)	Expected range
Ramachandran outliers	0.00 %	< 0.05%
Ramachandran favored	98.81 %	> 98%
Rotamer outliers	0.62 %	< 0.3%
C-beta deviations	73	0
Clashscore	4.87	(percentile:43.4)
Molprobity score	1.25	(percentile:66.4)
Cis-proline	33.33	0%
Cis-general	0.00	0%
