# Supplementary material for: Application of super-resolution and correlative double sampling in cryo-electron microscopy
Source: Faraday Discuss. 2022 Apr 22;240:261–76. doi: 10.1039/d2fd00049k (PMC9642007; doi:10.1039/d2fd00049k)
Supplement: FD-240-D2FD00049K-s002 [file FD-240-D2FD00049K-s002.pdf]

Electronic Supplementary Material (ESI) for Faraday Discussions.
This journal is © The Royal Society of Chemistry 2022

Supplementary Material

Sup Table 1 Previously published High resolution structures of apoferritin

Scope Detector Species	Mag	Pixel size Å	Total dose e/ Å2	N. of frames	N. of movies collected	N. of movies used	Defocus range	Picked particles	Particles used	Res Å
Krios F3 Human [3]	120K	0.492	50	40f (18.54s)	10398	*	0.3-1	1.47M	1.1M	1.25
Krios F3 Human [3]	120K	0.492	50	40f (18.54s)	10398	*	0.3-1	1.47M	800K	1.15
Krios Selectris F4 Mouse [5]	155K*	0.457	40	434eer (2s)	3370	3080		430K	360K	1.22
Krios Gif-K3 Human [4]	215K	0.4	45	30f (0.5s)	8034	6951	0.3-1.3	1.2M	900K	1.34
Krios F4 Human [4]	155K	0.502	40	40f (2s)	7734	5427	0.3-1.3	700K	500K	1.36
cryoARM K2 Mouse [13]	100K	0.49* or 0.3	32	*	*	*	0.3-3	120K	110K	1.34
Krios Gif-K3 CDS Mouse [10]	105K	0.834	69	120f (6s)	1035	*	0.3-0.7	*	570k	1.65
Krios Gif-K3 CDS Mouse [10]	64K	1.346	69	120f (15.6)	1032		0.3-0.7	*	373K	1.8
Krios G4 Gif-K3 Mouse [6]	215K	0.6	65	*EMDB	3472	*	0.2-0.8	*	185K	1.31
Falcon 4 no filter EER Mouse [6]	195K	0.6	50	*EMDB	3540	*	0.2-1	*	135K	1.43

Supplementary Figure Legends

Sup Fig 1 FSC curves tomography
FSC curves calculated with emClarity for the STA structures from the two-tilt series data set (A), six-tilt series data set (B), the 27-tilt series data set processed at non-SR (C) and SR (D).

Sup Fig 2 STA structures from 2-, 6-, and 27-tilt series data sets
A central slice through the STA structure of apoferritin from two-tilt series (A), six-tilt series STA apoferritin structure (B), 27-tilt series STA apoferritin structure (C), and the 27-tilt series SR STA apoferritin structure (D). The individual panels were generated using chimera.

Sup Fig 3 FSC curves SPA
FSC curves were calculated with Relion for the SPA structures from the 64K CDS SR data set (A), the 81K CDS SR data set (B), 130K CDS data set (C), and 130K EPU SR-bin2 (D).

Sup Fig 4 STA structure resolution comparisons
To demonstrate the features visible at different map resolutions, we compared a helix from residues 137-151 from the 27-tilt series non-SR STA reconstruction (A), SR STA reconstruction (B), the non-SR STA reconstruction calculated with cisTEM and sharpened in Relion (C), a 64K CDS SPA reconstruction from 13K particles (D), a 64K CDS SR SPA reconstruction from 54K particles (E) and the EMD-11603 STA reconstruction (F). The individual panels were generated using chimera.

Sup Fig 5 SPA non-SR reconstructions from 64K and 81K
The non-SR 64K SPA reconstruction with coordinates of apoferritin (top row A) and the non-SR 81K SPA apoferritin reconstruction with coordinates (bottom row B). Each of the panels is arranged with a helix from residues 137-151 shown on the left, a slice through the structure showing the water molecules in the middle, and the sodium binding site shown on the right. The individual panels were generated using chimera.

Sup Fig 6 Motion corrected summed movies and their power spectra in both non-SR and SR
A motion-corrected summed movie with its power spectra shown in non-SR and SR form 64K CDS data set (top row), the 81K data set (middle row), and the 130k data without the SR power spectra. The 2.1 Å reflections from the graphene layer on the grids are highlighted with red circles.

Sup Fig 7 SPA comparison between CDS SR and non-CDS non-SR
The 130K CDS SPA reconstruction with coordinates of apoferritin (top row A) and the 130K non CDS EPU-SRbin2 SPA apoferritin reconstruction with coordinates (bottom row B). Each of the panels is arranged with a helix from residues 137-151 shown on the left, a slice through the structure showing the water molecules in the middle, and the sodium binding site shown on the right. The individual panels were generated using chimera.

Sup Fig 8 B-factor plots
The B-factor plots were calculated from the 130K CDS SPA data sets (A), the 130K non CDS EPU-SR bin2 SPA data set (B), and the 64K CDS SR data set (C).

Sup Fig 9 Molprobity statistics for complete apoferritin complex
